# Optimal cutoff values for physical function tests in elderly patients with heart failure

**DOI:** 10.1038/s41598-022-10622-0

**Published:** 2022-04-28

**Authors:** Keita Aida, Kentaro Kamiya, Nobuaki Hamazaki, Kohei Nozaki, Takafumi Ichikawa, Takeshi Nakamura, Masashi Yamashita, Shota Uchida, Emi Maekawa, Jennifer L. Reed, Minako Yamaoka-Tojo, Atsuhiko Matsunaga, Junya Ako

**Affiliations:** 1 Department of Rehabilitation Sciences, Kitasato University Graduate School of Medical Sciences, Sagamihara, Japan; 2 Department of Physical Medicine and Rehabilitation, Saitama Medical Center, Jichi Medical University, Saitama, Japan; 3 Department of Rehabilitation, Kitasato University School of Allied Health Sciences, 1-15-1 Kitasato, Minami-ku, Sagamihara, Kanagawa 252-0375 Japan; 4 Department of Rehabilitation, Kitasato University Hospital, Sagamihara, Japan; 5 Department of Cardiovascular Medicine, Kitasato University School of Medicine, Sagamihara, Japan; 6 Exercise Physiology and Cardiovascular Health Lab, Division of Cardiac Prevention and Rehabilitation, University of Ottawa Heart Institute, Ottawa, Canada; 7 Faculty of Medicine, University of Ottawa, Ottawa, Canada; 8 Faculty of Health Sciences, School of Human Kinetics, University of Ottawa, Ottawa, Canada

**Keywords:** Cardiology, Geriatrics

## Abstract

Six-minute walk distance (6MWD) of 300 and 400 m are important targets of functional capacity. The present study was performed to determine cutoff values of physical function associated with 6MWD < 300 m and < 400 m in elderly patients with heart failure (HF). 6MWD, handgrip strength, quadriceps isometric strength (QIS), one-leg standing time (OLST), and 5-times sit-to-stand (5STS) before hospital discharge were evaluated in 1001 patients > 65 years (median age, 75: interquartile range, 71–80, 607 men) with HF. 6MWD < 300 and < 400 m were seen in 323 patients (32.3%) and 658 patients (65.7%), respectively. Handgrip strength, QIS, OLST, and 5STS were associated with 6MWD < 300 and < 400 m, respectively (*P* < 0.001). The cutoff values of handgrip strength, QIS, OLST, and 5STS were 18.9 kg, 35.0% body mass (BM), 9.1 s, and 9.5 s for 6MWD < 300 m, and 21.9 kg, 40.0% BM, 12.0 s, and 8.8 s for < 400 m, respectively. The cutoff values of physical function could be used to set cardiac rehabilitation goals and limiting determinants of reduced functional capacity in a clinical setting in elderly patients with HF.

## Introduction

Reduced functional capacity is a major consequence of heart failure (HF)^1^, and is associated with poor prognosis^2^, reduced activities of daily living (ADL)^3^, and poor quality of life^4^. Functional capacity is assessed by measuring the 6-minute walk distance (6MWD), and 6MWD < 300 m and < 400 m have been used as indicators of poor prognosis^5,6^ and mobility limitation^7,8^, respectively.

Physical function commonly decreases due to aging and sarcopenia in patients with cardiovascular diseases (CVD), especially HF^9^. Reduced functional capacity had been considered to result from impaired cardiac function. However, several studies suggested that patients with HF have significant skeletal muscle dysfunction and balance dysfunction that contribute to the associated reductions in functional capacity and ADL^10–12^. Therefore, it is important to assess physical function, including skeletal muscle function and balance function, in elderly patients with HF.

However, optimal cutoff values of physical function corresponding to 6MWD of 300 m and 400 m have yet to be determined in elderly patients with HF in a clinical setting. These cutoff values for physical function can be used in cardiac rehabilitation for training goals according to patients’ daily activity level needs. They are also useful to determine whether the limiting factor of functional capacity is physical function, such as muscle strength and balance function, or cardiopulmonary function.

The present study was performed to determine cutoff values of physical function associated with 6MWD < 300 m and < 400 m in elderly patients with HF.

## Materials and methods

### Study population and participants

This retrospective study was performed 1001 consecutive elderly patients ≥ 65 years old admitted to Kitasato University Hospital between May 2006 and March 2018 for acute HF, defined according to the Framingham criteria^13^, and participating in a cardiac rehabilitation program. We defined elderly according to the World Health Organization^14^.

We evaluated physical function (handgrip strength, quadriceps isometric strength [QIS], one-leg standing time [OLST], and 5-times sit-to-stand [5STS]) and functional capacity as 6MWD before hospital discharge. The study was performed in accordance with the tenets of the Declaration of Helsinki and the protocol was approved by the Ethics Committee of Kitasato University Medical Ethics Organization (KMEO B18-075). Because this study was an observational study that did not involve invasive procedures or interventions, written informed consent was not required under the Japanese Ministry of Health, Labor and Welfare’s "Ethical Guidelines for Medical and Health Research for Subjects". Therefore, informed consent was waived with Kitasato University Medical Ethics Organization approval by the institutional guidelines for retrospective observational studies. Participants were informed that they could refuse to participate because the study protocol was based on the retrospective review of medical records.

### Data collection

Data were obtained by electronic medical record review. Demographic data, causes of heart failure, biochemical data, echocardiogram, comorbidity, assessment of physical function (handgrip strength, QIS, OLST, and 5STS), and 6MWD were reviewed before hospital discharge. The B-type natriuretic peptide (BNP) concentration was measured using a commercially available immunoradiometric assay (Shionogi, Osaka, Japan). Estimated glomerular filtration rate (eGFR) was defined according to the formula created by the Japanese Society of Nephrology: 194 × (serum creatinine)^1.094^ × (age)^0.287^ (× 0.739 for women)^15^. Geriatric Nutritional Risk Index (GNRI), calculated based on serum albumin level and body mass index (BMI), was used as an indicator of nutritional status^16^. Left ventricular ejection fraction (LVEF) was estimated by applying Simpson’s method to two-dimensional echocardiograms. All tests were conducted prior to hospital discharge.

### Physical function and 6MWD measurement

Handgrip strength was measured using a digital dynamometer (TKK 5101 Grip-D; Takei, Tokyo, Japan). Maximal isometric voluntary contractions of the hands for 3 s each were collected for both hands with the elbow joint fixed at 90° in the sitting position. The highest value (expressed as the absolute value in kilograms) was used in the analysis.

Maximal QIS was measured with a handheld dynamometer (u-Tas; ANIMA, Tokyo, Japan) fixed to a rigid bar. Patients were asked to sit on a bench with their hip and knee flexed at an angle of 90°. The 5-s maximal isometric voluntary contractions of the quadriceps were collected three times successively for both legs. The participants were asked to gradually increase force to maximum voluntary effort and were told to avoid holding their breath during contractions to avoid the Valsalva maneuver. The greatest strength values on right and left sides were averaged and expressed as absolute values (kg) and relative to body mass (% BM).

As a measure of static body balance, the OLST was performed in which patients were required to stand on one leg for up to 60 s timed using a digital stopwatch with the eyes open. The test was performed twice. The timer was stopped if the patient made contact with any part of the room with any part of the body other than the supporting foot, began to hop around, or moved their hand off their hip. The highest value obtained was used in the analyses.

Functional muscle strength of the lower limbs was measured using the 5STS in which the time taken for patients to stand up and sit down five times without using their hands for support was recorded. The examination was terminated when the patient was unable to stand up straight.

The 6MWD was measured according to standard guidelines established by the American Thoracic Society^17^. Use of a walking aid during the test was permitted if required. Patients were instructed to walk, without running or jogging, covering as much distance as possible over a period of 6 min. Standardized encouragement was provided for patients every 1 min. After 6 min, patients were instructed to stop walking and the distance walked was recorded.

### Statistical analysis

Continuous variables are expressed as the median (interquartile range), and categorical variables are expressed as the number (%). Multivariate normal imputation for missing values was performed using JMP Pro 14.2 (SAS Institute Inc., Cary, NC)^18^. The following variables were incorporated into the imputation model: age, sex, height, body mass, heart rate, systolic blood pressure (SBP), current smoker, LVEF, New York Heart Association (NYHA) functional class, GNRI, hemoglobin, eGFR, log BNP, prior HF, handgrip strength, QIS, OLST, and 5STS.

The adjusted odds ratios (ORs) and 95% confidence intervals (95% CIs) were calculated by univariate and multivariate logistic regression analyses. To identify the clinical predictors of 6MWD < 300 m and 6MWD < 400 m, multivariate models were adjusted for age, sex, height, body mass, heart rate, SBP, current smoker, NYHA functional class, LVEF, GNRI, hemoglobin, eGFR, log BNP, and prior HF. The unit changes in each variable were 1 kg for handgrip strength, 1% BM for QIS, and 1 s for OLST and 5STS.

Nonlinear associations between 6MWD and parameters of physical function, i.e., handgrip strength, QIS, OLST, and 5STS, were modeled using restricted cubic splines with four knots.

Handgrip strength, QIS, OLST, and 5STS cutoff values for 6MWD (300 m^5,6^ and 400 m^7,8^) were determined using the area under the curve (AUC) of the receiver-operating-characteristic (ROC) curve. The optimal cutoff values for handgrip strength, QIS, OLST, and 5STS was defined as the values closest to the northwest point of the ROC curves. We compared the AUCs for handgrip strength and lower extremity physical function parameters (QIS, OLST, and 5STS). We compared the AUCs for handgrip strength and lower extremity physical function parameters (QIS, OLST, and 5STS). We evaluated the incremental predictive performance of 6MWD < 300 and < 400 m considering lower extremity physical function, AUC, net reclassification improvement (NRI), and integrated discrimination improvement (IDI). Sensitive statistical methods to quantify the model’s improvement were constructed by adding the lower extremity physical function parameters (QIS, OLST, and 5STS) to the handgrip strength^19,20^.

Statistical analyses were performed with Stata version 16.1 (Stata Corp, College Station, TX) and JMP Pro 14.1 (SAS Institute Inc.). In all analyses, a two-tailed *P* < 0.05 was taken to indicate statistical significance.

### Ethics approval

The study was performed in accordance with the tenets of the Declaration of Helsinki and the protocol was approved by the Ethics Committee of Kitasato University Medical Ethics Organization.

## Results

### Patient characteristics

Baseline characteristics for all participants are shown in Table 1. The median age of the study population was 75 (range 71–80) years, 60.6% of the patients were male, and 39.7% had reduced LVEF. The study population had a median handgrip strength of 20.7 kg (range 16.0–26.1), OLST of 9.9 s (range 4.1–26.1), QIS of 37.6% BM (range 29.9–45.6), 5STS of 9.2 s (range 7.6–11.8), and 6MWD of 360 m (range 270–422).

**Table 1:** Baseline characteristics.

	Overall
	(*n* = 1001)
Age, yrs	75 [71, 80]
Male	607 (60.6)
Height, cm	159.0 [152.0, 165.0]
Body mass, kg	53.5 [46.8, 61.5]
BMI, kg/m^2^	21.3 [19.2, 23.6]
Heart rate, beats/min	76 [67, 88]
SBP, mm Hg	115 [101, 133]
DBP, mm Hg	63 [55, 74]
Current smoker, %	106 (10.6)
**LVEF, %**	50.0 [35.9, 62.7]
LVEF < 40	394 (39.4)
LVEF 40–49	112 (11.1)
LVEF ≥ 50	495 (49.5)
**Causes of heart failure, %**	
Ischemic heart disease	385 (38.5)
Valvular heart disease	207 (20.7)
Cardiomyopathy	131 (13.1)
Other	278 (27.8)
**NYHA functional class, %**	
II	641 (64.0)
III	202 (20.2)
IV	2 (0.2)
**Comorbidity, %**	
Hypertension	759 (75.8)
Diabetes	483 (48.3)
Dyslipidemia	460 (46.0)
Prior myocardial infarction	178 (17.8)
Prior heart failure admission	489 (48.9)
**Laboratory data**	
Albumin, g/dL	3.5 [3.2, 3.9]
GNRI	93.2 [86.7, 100.2]
BNP, pg/mL	331.85 [155.12, 721.53]
log BNP, pg/mL	5.8 [5.0, 6.6]
eGFR, mL/min/1.73 m^2^	45.7 [30.1, 58.5]
Hemoglobin, g/dL	11.7 [10.3, 13.2]
**Physical function**	
Handgrip strength, kg	20.7 [16.0, 26.1]
OLST, s	9.9 [4.1, 26.1]
QIS, % BM	37.6 [29.9, 45.6]
5STS, s	9.2 [7.6, 11.8]
6MWD, m	360 [270, 422]
< 300	323 (32.3)
< 400	658 (65.7)

Values are the median [interquartile range] or number (%).

% BM, percentage of body mass; 5STS, 5-times sit-to-stand; 6MWD, 6-minute walk distance; BMI, body mass index; BNP, B-type natriuretic peptide; DBP, diastolic blood pressure; eGFR, estimated glomerular filtration rate; GNRI, Geriatric Nutritional Risk Index; LVEF, left ventricular ejection fraction; NYHA, New York Heart Association; OLST, one-leg standing time; QIS, quadriceps isometric strength.; SBP, systolic blood pressure.

### Relationship between functional capacity and physical function

**Figure 1:**
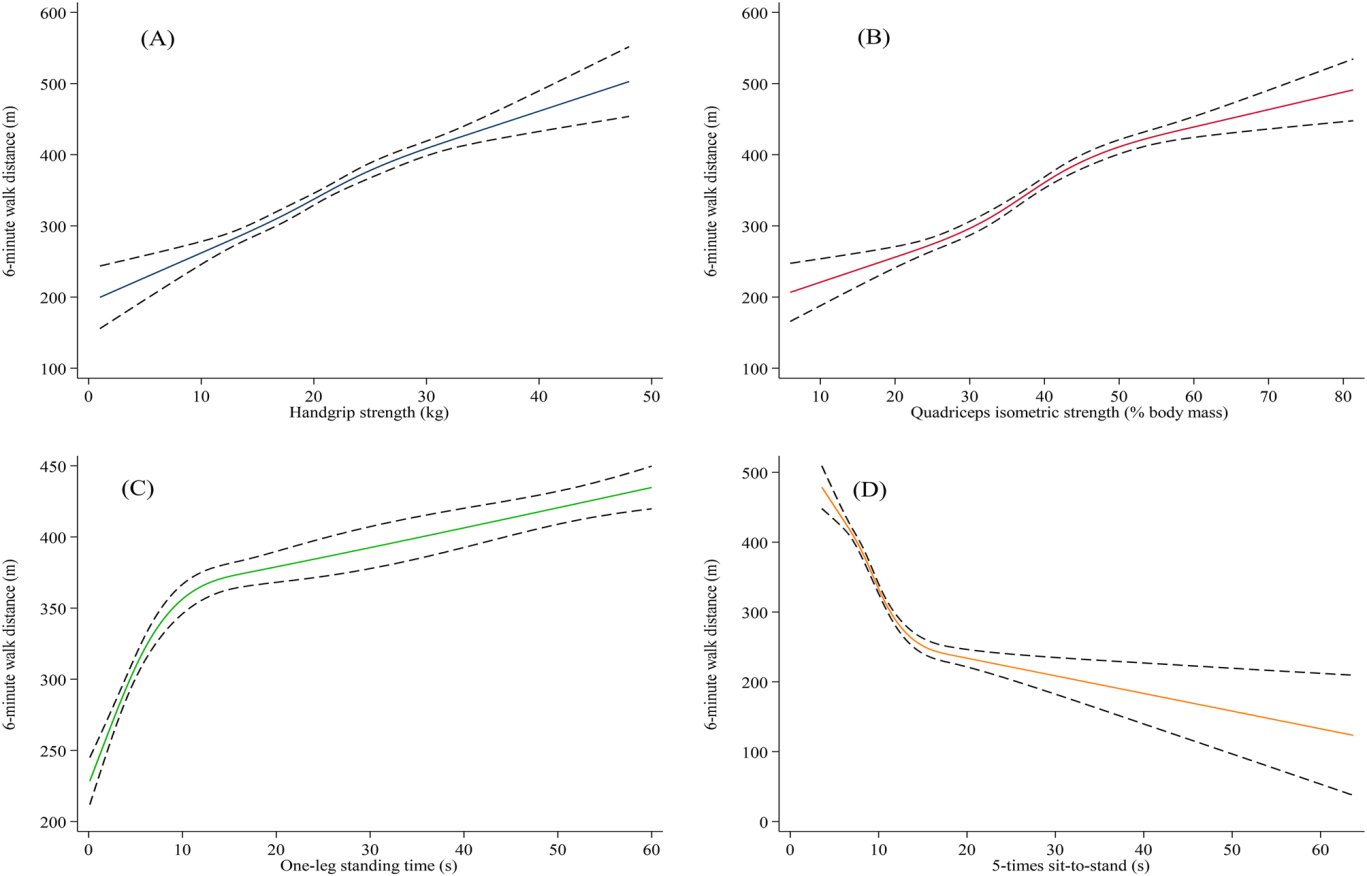
Associations of 6-minute walk distance with handgrip strength (**A**), quadriceps isometric strength (**B**), one-leg standing time (**C**), and 5-times sit-to-stand (**D**). Dotted lines indicate the 95% confidence intervals.

Overall, 323 patients (32.3%) and 658 patients (65.7%) had 6MWD < 300 m and 6MWD < 400 m before hospital discharge, respectively. The results of univariate and multivariate logistic regression analyses for 6MWD < 300 m and 6MWD < 400 m are shown in Table 2. All physical function measures were still factors independently associated with reduced functional capacity, even after adjusting for age, sex, height, body mass, heart rate, SBP, current smoker, NYHA functional class, LVEF, GNRI, hemoglobin, eGFR, log BNP, and prior HF. The associations between 6MWD and parameters of physical function are shown using the restricted cubic spline procedure in Fig. 1. 6MWD increased with increasing handgrip strength, QIS, and OLST, and decreasing 5STS.

**Table 2:** Univariate and multivariate logistic regression for 6-minute walk distance < 300 m and 6-minute walk distance < 400 m.

	6MWD < 300 m						6MWD < 400 m					
	Univariate			Multivariate			Univariate			Multivariate		
	OR	95% CI	*P*-value	OR	95% CI	*P*-value	OR	95% CI	*P*-value	OR	95% CI	*P*-value
Handgrip strength per 1 kg	0.89	0.87–0.91	< 0.001	0.91	0.88–0.95	< 0.001	0.88	0.86–0.90	< 0.001	0.88	0.85–0.91	< 0.001
Quadriceps isometric strength per 1% BM	0.90	0.89–0.92	< 0.001	0.92	0.90–0.94	< 0.001	0.91	0.90–0.92	< 0.001	0.93	0.91–0.94	< 0.001
One-leg standing time per 1 s	0.92	0.91–0.94	< 0.001	0.95	0.94–0.97	< 0.001	0.96	0.95–0.96	< 0.001	0.97	0.96–0.98	< 0.001
5-times sit-to-stand per 1 s	1.33	1.28–1.40	< 0.001	1.22	1.16–1.30	< 0.001	1.56	1.45–1.68	< 0.001	1.53	1.41–1.66	< 0.001

*Adjusted for age, sex, height, body mass, heart rate, SBP, current smoker, NYHA functional class, LVEF, GNRI, hemoglobin, eGFR, log BNP, and prior heart failure for 6MWD < 300 m and < 400 m.

6MWD, 6-minute walk distance; BNP, B-type natriuretic peptide; % BM, percentage of body mass; CI, confidence intervals; eGFR, estimated glomerular filtration; GNRI, geriatric nutritionl risk index; LVEF; left ventricular ejection fraction; NYHA, New York Heart Association; left ventricular ejection fraction; OR, odds ratio; SBP, systolic blood pressure.

### Cutoff values of physical function measures for 6MWD < 300 m and < 400 m

**Figure 2:**
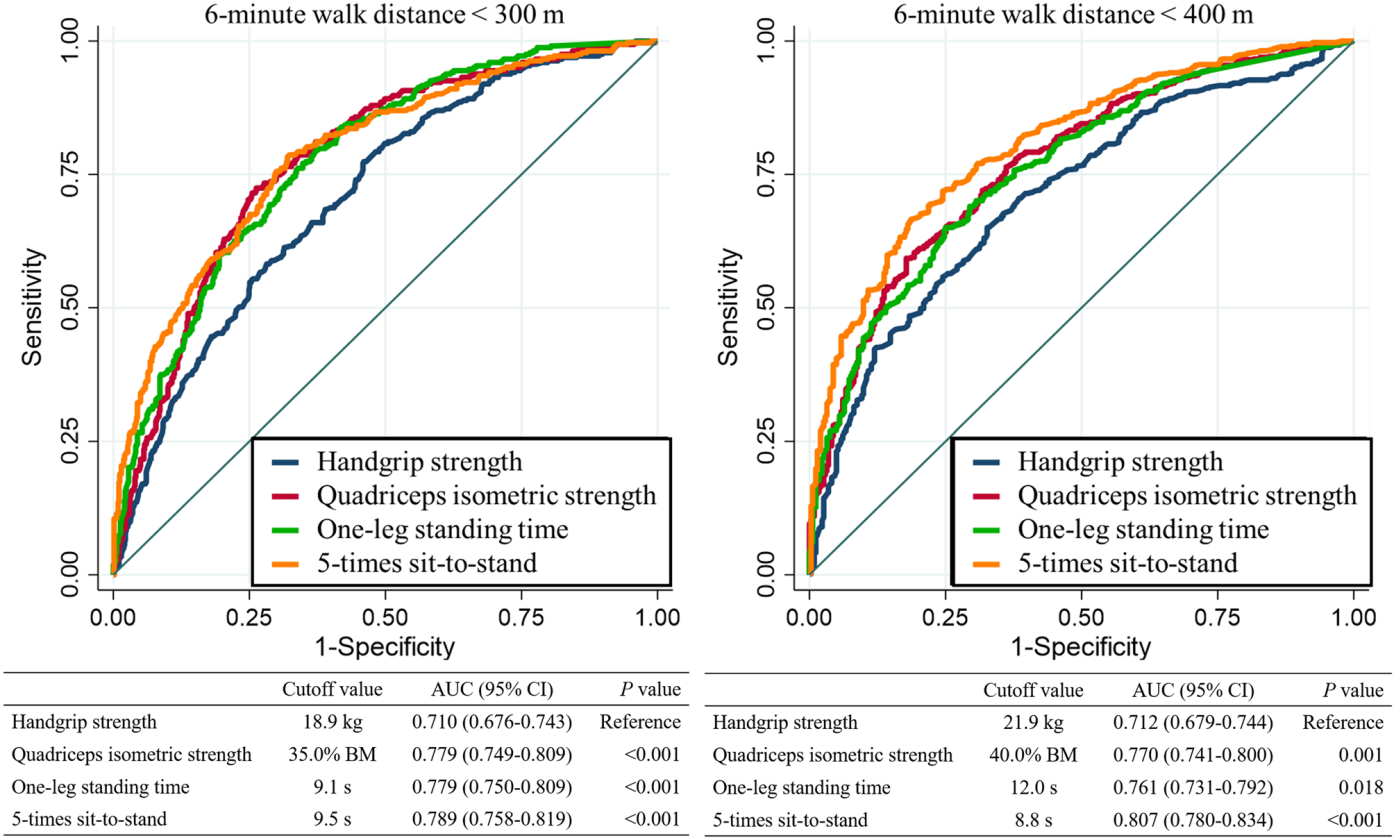
Receiver operating characteristic curve analysis for predicting reduced functional capacity (6-minute walk distance < 300 m and < 400 m) according to handgrip strength, quadriceps isometric strength, one-leg standing time, and 5-times sit-to-stand. 6MWD, 6-minute walk distance; AUC, area under the curve; CI, confidence intervals.

Figure 2 shows the ROC curves for handgrip strength, QIS, OLST, and 5STS as predictors of 6MWD < 300 m and < 400 m. The AUCs for handgrip strength, QIS, OLST, and 5STS were 0.710 (95% CI 0.676–0.743), 0.779 (95% CI 0.749–0.809), 0.779 (95% CI 0.750–0.809), and 0.789 (95% CI 0.758–0.819) for 6MWD < 300 m, respectively; and 0.712 (95% CI 0.679–0.744), 0.770 (95% CI 0.741–0.800), 0.761 (95% CI 0.731–0.792), and 0.807 (95% CI 0.780–0.834) for 6MWD < 400 m, respectively. Using ROC analysis of 6MWD < 300 m and < 400 m, we identified cutoff values for handgrip strength of 18.9 kg and 21.9 kg, with sensitivity of 0.685 and 0.673, and specificity of 0.613 and 0.650, respectively. The cutoff values for QIS were 35.0% BM and 40.0% BM, with sensitivity of 0.724 and 0.720, and specificity of 0.737 and 0.682, respectively. The cutoff values for OLST were 9.1 s and 12.0 s, with sensitivity of 0.759 and 0.690, and specificity of 0.663 and 0.705, respectively. The cutoff values for 5STS were 9.5 s and 8.8 s, with sensitivity of 0.777 and 0.719, and specificity of 0.680 and 0.755, respectively.

We compared the AUCs for lower extremity physical function (QIS, OLST, and 5STS) and handgrip strength (Fig. 2). Furthermore, we evaluated the incremental predictive performance of the lower extremity physical function parameters for 6MWD < 300 and < 400 m. To do so, we compared the AUC, NRI, and IDI between handgrip strength plus lower extremity physical function parameters (QIS, OLST, and 5STS) for 6MWD < 300 m and < 400 m. The AUC, NRI, and IDI analyses indicated that lower extremity physical function parameters (QIS, OLST, and 5STS) provided additional predictive performance beyond handgrip strength for 6MWD < 300 m and < 400 m (supplement Table S1). The combined parameters of lower extremity physical function (handgrip strength, QIS, OLST, and 5STS) showed significantly greater discriminative capabilities for reduced functional capacity than handgrip strength alone. The parameters of lower extremity physical function, i.e., handgrip strength, QIS, OLST, and 5STS showed significantly greater discriminative capabilities for reduced functional capacity than handgrip strength (Fig. 2).

## Discussion

The primary findings of the present study were as follows. First, the results indicated that physical function evaluated based on muscle strength and balance function were associated with 6MWD in elderly patients with HF. Second, the following physical function values were selected as optimal cutoff points for 6MWD < 300 m and < 400 m: handgrip strength, 18.9 kg and 21.9 kg, respectively; QIS, 35.0% BM and 40.0% BM, respectively; OLST, 9.1 s and 12.0 s, respectively; and 5STS, 9.5 s and 8.8 s, respectively.

### Physical function and functional capacity in heart failure patients

There have been several reports on factors that define 6MWD in patients with heart failure. Many reports indicated the associations between 6MWD and general clinical background factors, and factors related to cardiac function and hemodynamics^11,21^. On the other hand, 6MWD has been reported to show an extremely high correlation with normal walking speed over short distances in elderly patients with heart disease^22^. Many studies have shown that muscle strength and balance function are usually major determinants of walking speed in elderly patients^23,24^. Taken together, these observations along with the results of the present study suggest that 6MWD in elderly heart failure patients may also be strongly affected by determinants of physical function, such as muscle strength and balance function.

### Cutoff values of parameters of physical function in heart failure patients

A previous study indicated that QIS is associated with exercise capacity, expressed as estimated metabolic equivalent (eMET) categories, in patients with coronary artery disease. This study showed that QIS cutoff values of 40%, 50%, and 60% BM can predict exercise capacity levels of 5, 7, and 10 eMETs, respectively^25^. In the present study, QIS of 35% and 40% BM, which predicted 6MWD < 300 m and < 400 m, approximated the results of this previous study, suggesting that they may contribute to target cutoff values of functional capacity in clinical settings.

A previous study indicated that a handgrip strength cutoff point of 32.5 kg was equivalent to exercise intolerance in male outpatients with HF^26^. Unlike this previous study, the present study showed cutoff values of 18.9 and 21.9 kg for handgrip strength in inpatients with 6MWD < 300 m and < 400 m, respectively, which were approximately 60%–70% of the values in the previous study, because the patients were younger, only male, and the study population was smaller (148 vs. 1001 in the present study) than in the present study. Therefore, cutoff values of 18.9 and 21.9 kg for handgrip strength in inpatients with 6MWD < 300 m and < 400 m may, respectively, reflect the results for older HF patients of both sexes in a large cohort.

A previous study indicated that a cutoff value of 3 s for OLST is associated with high mortality risk in patients ≥ 75 years old with CVD^12^. In the present study, cutoff values of OLST for reduced functional capacity of 6MWD < 300 m and < 400 were 9.1 s and 12.0 s, respectively, suggesting that they may contribute to the target cutoff values for reduced functional capacity in clinical settings.

Several studies showed that cutoff values of 5STS < 6.25 s and 13 s can predict 6MWD < 350 m in patients with COPD^27,28^. However, the cutoff value of 5STS remains controversial. To our knowledge, this is the first report that 5STS cutoff values of 9.5 s and 8.8 s can be used to predict reduced functional capacity, i.e., 6MWD < 300 m and < 400 m, respectively, in elderly patients with HF.

### Utility of determining lower extremity physical function compared to handgrip strength

The results of the present study indicated that lower extremity physical function showed significantly greater ability to predict reduced functional capacity than handgrip strength in elderly patients with HF. Reduced handgrip strength and QIS were reported to be associated with exercise intolerance in outpatients with HF^29^. However, there have been no reports of direct comparisons of the predictive capabilities of handgrip strength and lower extremity physical function for reduced functional capacity. Recent studies showed that lower extremity physical function was more clinically useful to predicting ADL, instrumental ADL, mobility disability, and physical activity than grip strength in community-dwelling elderly men and nursing home residents^30,31^. Additionally, lower extremity physical function are related to functional capacity in patients with HF^11,32^. The reason for these results may be that the 6-minute walk test is an exercise of the lower limbs, and the muscular endurance, strength, and balance functions of the lower limbs were directly affected. These findings may explain the relationship in the present study. Therefore, the results of the present study suggested that lower extremity physical function is more clinically relevant for predicting reduced functional capacity than handgrip strength in elderly patients with HF.

### Mechanism underlying the association between reduced physical function and reduced functional capacity

Several possible mechanisms may underlie the observed association between physical function and functional capacity in hospitalized patients with HF. HF is characterized by fatigue and dyspnea^33^ both of which lead to reduced physical activity. Reduced physical activity promotes skeletal muscle deconditioning^34^ and may result in further reductions in physical function. On the other hand, some reports suggested that patients with HF have structural and/or functional skeletal muscle abnormalities^35^. Further, several studies demonstrated that patients with HF showed loss of skeletal muscle mass^36^, shift from slow to fast twitch fibers^37^, and mitochondrial dysfunction^38^. Reduced skeletal muscle strength itself was suggested to result in a decrease in physical function^39^. All of the factors outlined above can result in decreases in the level of physical function, which may be related to reduced functional capacity in HF patients.

### Clinical implications

The findings of the present study indicated that cutoff values of parameters of physical function may be used to determine the goals of cardiac rehabilitation and limiting determinants of reduced functional capacity in clinical settings in elderly patients with HF. The methods of physical assessment used in this study are convenient and can be used repeatedly and rapidly without the need for expensive equipment or specialized facilities to determine the physical condition of elderly HF patients. Several recent studies showed that about half of all inpatients had physical frailty^40,41^. Handgrip strength, QIS, OLST, and 5STS could be useful means of estimating the functional capacity of frail patients who cannot directly perform the 6MWD test due to their general condition or in settings without the availability of a long walking track.

## Limitations

The present study had several limitations. First, this was a single-center, retrospective study performed in Japan. Second, patients who could not perform the physical function and 6MWD tests were excluded, which may have resulted in bias. Third, we did not have data regarding potential confounding factors, such as pulmonary function and skeletal muscle mass. Finally, the study population included only Asian subjects, so the generalizability of the results to other ethnicities remains unclear.

## Conclusion

In summary, the results of the present study suggested that reduced physical function, particularly lower extremity physical function, is associated with reduced functional capacity in elderly patients with HF. In addition, the cutoff values for handgrip strength of 18.9 kg and 21.9 kg, QIS of 35% BM and 40% BM, OLST of 9.1 s and 12.0 s, and 5STS of 9.5 s and 8.8 s for 6MWD 300 m and 400 m, respectively. These cutoff values for physical function can be used in cardiac rehabilitation for the definition of training goals according to patients’ needs with regard to ADL, and also to determine whether the limiting factor of functional capacity is physical function, such as muscle strength and balance function, or cardiopulmonary function.

## Supplementary Information

Supplementary Information.

## Abbreviations

ADL: Activities of daily living
AUC: Area under the curve
BM: Body mass
BMI: Body mass index
BNP: B-type natriuretic peptide
CI: Confidence intervals
CVD: Cardiovascular disease
GNRI: Geriatric nutritional risk index
HF: Heart failure
IDI: Integrated discrimination improvement
LVEF: Left ventricular ejection fraction
NRI: Net reclassification improvement
OR: Odds ratio
OLST: One-leg standing time
QIS: Quadriceps isometric strength
SBP: Systolic blood pressure
5STS: 5-times sit-to-stand
6MWD: 6-minute walk distance
