# Supplementary material for: Optimal cutoff values for physical function tests in elderly patients with heart failure
Source: Sci Rep. 2022 Apr 28;12:6920. doi: 10.1038/s41598-022-10622-0 (PMC9051131; doi:10.1038/s41598-022-10622-0)
Supplement: Supplementary file 1 — Supplementary Information. [file 41598_2022_10622_MOESM1_ESM.pdf]

Title: Optimal cutoff values for physical function tests in elderly patients with heart failure

Keita Aida, Kentaro Kamiya, Nobuaki Hamazaki, Kohei Nozaki, Takafumi Ichikawa, Takeshi Nakamura, Masashi Yamashita, Shota Uchida, Emi Maekawa, Jennifer L Reed, Minako Yamaoka-Tojo, Atsuhiko Matsunaga, Junya Ako

Supplemental Table S1. The incremental predictive performance for 6-minute walk distance < 300 m and 6-minute walk distance < 400 m

**6-minute walk distance < 300 m**	AUC	95% CI	*P* -Value	NRI	95% CI	*P* -Value	IDI	95% CI	*P* -Value
Handgrip strength	0.710	0.676 - 0.743	reference			reference			reference
+ Quadriceps isometric strength	0.786	0.757 - 0.815	<0.001	0.670	0.525 - 0.805	<0.001	0.099	0.064 - 0.139	<0.001
+ One-leg standing time	0.795	0.767 - 0.824	<0.001	0.712	0.588 - 0.839	<0.001	0.106	0.076 - 0.145	<0.001
+ 5-times sit-to-stand	0.817	0.788 - 0.845	<0.001	0.715	0.595 - 0.858	<0.001	0.156	0.104 - 0.208	<0.001
Handgrip strength	0.710	0.676 - 0.743	reference			reference			reference
+ Quadriceps isometric strength + One-leg standing time	0.823	0.796 - 0.850	<0.001	0.846	0.703 - 0.969	<0.001	0.160	0.120 - 0.211	<0.001
+ Quadriceps isometric strength + 5-times sit-to-stand	0.837	0.810 - 0.864	<0.001	0.891	0.744 - 1.009	<0.001	0.189	0.139 - 0.242	<0.001
+ One-leg standing time + 5-times sit-to-stand	0.842	0.816 - 0.868	<0.001	0.836	0.701 - 0.973	<0.001	0.203	0.153 - 0.255	<0.001
Handgrip strength	0.710	0.676 - 0.743	reference			reference			
+ Quadriceps isometric strength + One-leg standing time + 5-times sit-to-stand	0.852	0.827 - 0.877	<0.001	0.944	0.805 - 1.096	<0.001	0.223	0.174 - 0.276	<0.001

**6-minute walk distance < 400 m**	AUC	95% CI	*P* -Value	NRI	95% CI	*P* -Value	IDI	95% CI	*P* -Value
Handgrip strength	0.712	0.679 - 0.744	reference			reference			reference
+ Quadriceps isometric strength	0.785	0.679 - 0.744	<0.001	0.630	0.496 - 0.750	<0.001	0.116	0.081 - 0.155	<0.001
+ One-leg standing time	0.784	0.754 - 0.813	<0.001	0.608	0.498 - 0.738	<0.001	0.102	0.067 - 0.146	<0.001
+ 5-times sit-to-stand	0.833	0.807 - 0.860	<0.001	0.880	0.759 - 1.005	<0.001	0.179	0.116 - 0.241	<0.001
Handgrip strength	0.712	0.679 - 0.744	reference			reference			reference
+ Quadriceps isometric strength + One-leg standing time	0.813	0.787 - 0.840	<0.001	0.726	0.616 - 0.869	<0.001	0.158	0.121 - 0.207	<0.001
+ Quadriceps isometric strength + 5-times sit-to-stand	0.846	0.820 - 0.873	<0.001	0.933	0.815 - 1.055	<0.001	0.214	0.161 - 0.272	<0.001
+ One-leg standing time + 5-times sit-to-stand	0.850	0.825 - 0.876	<0.001	0.999	0.843 - 1.104	<0.001	0.216	0.157 - 0.279	<0.001
Handgrip strength	0.712	0.679 - 0.744	reference			reference			reference
+ Quadriceps isometric strength + One-leg standing time + 5-times sit-to-stand	0.856	0.831 - 0.882	<0.001	0.989	0.866 - 1.122	<0.001	0.235	0.185 - 0.296	<0.001

AUC indicates area under the receiver operating characteristic curve; CI, Confdence interval; NRI, net reclassification improvement; and IDI, integrated discrimination improvement.
